# Anesthetic Considerations on Adrenal Gland Surgery

**DOI:** 10.14740/jocmr1960w

**Published:** 2014-10-16

**Authors:** Rudin Domi, Hektor Sula, Myzafer Kaci, Sokol Paparisto, Artan Bodeci, Astrit Xhemali

**Affiliations:** a Department of Anesthesiology and Intensive Care Medicine, “Mother Teresa” University Hospital Center, Tirana, Albania; b Department of General Surgery, “Mother Teresa” University Hospital Center, Tirana, Albania; c Department of Oncologic Surgery, “Mother Teresa” University Hospital Center, Tirana, Albania

**Keywords:** General anesthesia, Adrenal gland, Preoperative evaluation

## Abstract

Adrenal gland surgery needs a multidisciplinary team including endocrinologist, radiologist, anesthesiologist, and surgeon. The indications for adrenal gland surgery include hormonal secreting and non-hormonal secreting tumors. Adrenal hormonal secreting tumors present to the anesthesiologist unique challenges requiring good preoperative evaluation, perioperative hemodynamic control, corrections of all electrolytes and metabolic abnormalities, a detailed and careful anesthetic strategy, overall knowledge about the specific diseases, control and maintaining of postoperative adrenal function, and finally a good collaboration with other involved colleagues. This review will focus on the endocrine issues, as well as on the above-mentioned aspects of anesthetic management during hormone secreting adrenal gland tumor resection.

## Introduction

The adrenal cortex produces three types of hormones: glucocorticoids (cortisol), mineralocorticoids (aldosterone and 11-deoxycorticosterone), and androgens. Cushing’s syndrome caused either by the overproduction of cortisol by the adrenal cortex or exogenous glucocorticoid therapy, results in a syndrome characterized by truncal obesity, hypertension, hyperglycemia, increased intravascular fluid volume, hypokalemia, abdominal striae, osteoporosis, and muscle weakness. Aldosterone is a major regulator of extracellular volume and potassium homeostasis. Hypersecretion (Conn’s syndrome) of the major adrenal mineralocorticoid aldosterone increases the renal tubular exchange of sodium for potassium and hydrogen ions, leading to hypertension, hypokalemic alkalosis, skeletal muscle weakness, and fatigue. Adrenal medulla produces epinephrine and norepinephrine. Pheochromocytoma is a neuroendocrine tumor arising from chromaffin cells in the adrenal medulla causing less than 0.1% of all cases of hypertension. Usually, tumors are benign but in 10% of the cases they may be malignant. These tumors may secrete the catecholamine (dopamine, norepinephrine, and epinephrine). Indications for adrenalectomy include primary or secondary (metastasis) tumors of adrenal glands and of course hormonal secretion diseases like Cushing (glucocorticoid excess), Conn (mineral corticoid excess), and pheochromocytoma (catecholamine excess). This review is focused on anesthetic management of these diseases.

## Conn’s Syndrome

### General considerations

Hyperaldosteronism is characterized by an excess of aldosterone. Hyperaldosteronism can be divided in primary and secondary one. The secondary hyperaldosteronism may be due to severe liver diseases, nephritic syndrome, and cardiac failure. The primary form (Conn’s syndrome) presents the excess of aldosterone due to an adrenal gland disease. In the majority of cases (60%) a unilateral adenoma may be verified, whereas bilateral adrenal hyperplasia is faced in 30% of cases. It is more common in females than males [1, 2], and is the cause of 5-13% of secondary hypertension and less than 1% of essential one [2]. As aforementioned hyperaldosteronism can be two forms: primary and secondary hyperaldosteronism. Primary hyperaldosteronism is known as Conn’s syndrome resulting from unilateral or bilateral adrenal gland tumors [3-5]. Secondary hyperaldosteronism is due to increased levels of renin, inducing the renin-aldosterone axis activitation. These pathophysiologic changes are usually present in severe cardiac failure, nephritic syndrome, and advanced liver disease. The clinical features include systemic hypertension, metabolic alkalosis, hypokalemia, increased urinary excretion of potassium, hypernatremia, fatigue, muscle cramps, and skeletal muscle weakness. Systemic hypertension (often increased diastolic pressure) can be result of aldosterone-induced sodium and water retention. This hypertension is often resistant of pharmacologic treatment. Fatigue, muscle cramps, and muscle weakness are presented due to hypokalemia. Diagnosing Conn’s syndrome is quite simple. Clinical features can suggest the diagnosis but are not specific, but Conn’s syndrome is suspected in presence of diastolic hypertension, low plasma renin and high plasma aldosterone not able to be suppressed by fluid challenge [6]. After this scenario the imagining methods and biochemistry examination are of great importance. Biochemistry examinations include renin and aldosterone blood level, potassium and sodium plasma concentration. Increased renin level can be faced in secondary hyperaldosteronism, whereas decreased renin is usually found in Conn’s syndrome, associated with high aldosterone level. Aldosterone increases the effect of catecholamines, due to noradrenaline re-uptake blocking effect, and it predisposes to myocardial fibrosis resulting in arrhythmias and myocardial ischemia [7-9]. Hypokalemia is a constant finding, whereas hypernatremia can commonly be faced. Imagining examinations include ultrasound, angio-CT scan, and MRI. The diagnosis was confirmed by clinical features, and biochemistry findings, and supported by imagining examinations.

### Anesthetic considerations

The anesthesiologist must deal with intraoperative hemodynamic changes and hypokalemia. It is well-known that hypokalemia and metabolic alkalosis may prolong the action of neuromuscular blocking agents, inducing bradycardia. Hypokalemia may be worsened by respiratory alkalosis (hyperventilation) and by sevoflurane-induced polyuria. The anesthesiologist must avoid hyperventilation and sevoflurane use as an inhalation anesthetic drug. The manipulation of adrenal gland during dissection and resection may lead to catecholamine release from the adrenal medulla with resultant hemodynamic fluctuations [9, 10]. It is recently reported that Conn’s syndrome can produce brisk and untreatable intraoperative hypertension [11]. The preoperative fluid volume status evaluation (the presence of orthostatism, increased heart rate, blood pressure, increased hematocrite, etc.) can detect hypovolemia. Hypovolemia is a rare condition and can be multifactorial including diuretics’ use, anesthetic drugs’ effects, positive pressure ventilation, laparoscopic approach, and the patient’s position. Both hypervolemia and hypovolemia must be aggressively treated. Laparoscopic approach remains gold standard [12, 13]. It is generally accepted that laparoscopic approach may decrease postoperative cardiac and respiratory complications, less postoperative pain, and early ambulation [14]. Supplementation of hydrocortisone is another issue that anesthesiologist must deal with. Cortisol administration is helpful in perioperative hypoadrenocorticism or in chronic steroid administration [15]. Adrenal suppression induces hypotension, decreased cardiac output, hyponatremia, and hypoglycemia. It is mandatory to control cortisol level in preoperatively inadequate cortisol secretion patient, and cortisol supplementation as well. Etomidate must be avoided because it interferes with cortisol synthesis [16]. As a conclusion, Conn’s syndrome presents different problems to the anesthesiologist. The anesthesiologist must deal with hypertension, hypervolemia, hypokalemia, and depending case by case with cortisol supplementation. A good cooperation between the anesthesiologist, endocrinologist, and surgeon is strongly recommended. The hottest problems are presented in Table 1.

**Table 1:** Conn’s Syndrome and Anesthetic Management

Conn’s syndrome	Treatment
Preoperative period	
Preoperative hypokalemia	Begin spironolactone, supplement kalium
Hypertension	Continue preoperative antihypertensive drugs
Premedication	Adequate sedation
Intraoperative period	
Metabolic alkalosis	Avoid hyperventilation
Hemodynamic state	Strict hemodynamic control
Potassium level, gas analysis	Frequent measures of acid-base status and potassium blood level

## Cushing’s Syndrome

### General considerations

Cushing’s syndrome presents a typical complexity of clinical features, due to excessive circulating glucocorticoid level. The increased glucocorticoid level can be from either endogenous oversecretion or chronic treatment with glucocorticoids at higher doses. Approximately 70% of endogenous Cushing’s syndrome are due to Cushing’s disease (a primary pituitary ACTH-producing tumor), 15% results from ectopic production of ACTH, and the last 15% are secondary to an adrenal tumor. It has been recently reported that Cushing’s syndrome can rarely be caused by administration of oral steroids, injections of steroids, inhalers and unguents as well [17]. Patients under chronic steroid therapy (allergies, asthma, and arthritis) may develop Cushing’s syndrome. Several authors reported Cushing’s syndrome associated with other tumors or clinical situations as pheochromocytoma [18, 19], sarcoidosis [20], pancreatic acinar cell carcinoma [21], pre-eclamptic findings [22], malignant gastrinoma [23], bronchial carcinoid lung tumor [24], pancreatic neuroendocrine tumor and Hippel-Lindau disease [25], and mesenteric neuroendocrine carcinoma [26].

The clinical scenario of Cushing’s syndrome is a very characteristic one. Because of weight gain, the patients present the typical trio “moon facies”, “buffalo hump”, and central obesity. Skin thining and bruising associated with purple striae is another physical sign. Clinical manifestations also include proximal muscle weakness, fatigue, and spontaneous bone fractures due to osteopenia, sexual disorders (amenorrhea, infertility, menstrual irregularities, and decreased libido), hypertension secondary to water retention, hyperglycemia, metabolic alkalosis, and hypokalemia. The clinical features are summarized in Table 2 [14]. Cushing’s disease resulting from pituitary adenoma can be manifested also with visual disturbances, head-ache, and elevated intracranial pressure.

**Table 2:** Clinical Manifestation of Cushing’s Syndrome [14]

Central obesity	Thin extremities
Supraclavicular fat	Proximal muscle weakness
Moon face	Hypertension
Buffalo hump	Hyperglycemia
Abdominal striae	Metabolic alkalosis
Skin thinning	Hypokalemia
Easy bruising	Menstrual irregularities
Osteopenia	Poor wound healing

Generally the patient suffering from Cushing’s syndrome is evaluated by the endocrinologist and then referred to the surgical team. The diagnosis is usually confirmed by imagery and laboratory tests. Increased blood and urinary cortisol level, elevated urinary 17-hydroxycorticosteroids, and excessive plasma ACTH level, can suggest the diagnosis [27]. The next step is to differentiate Cushing’s syndrome by Cushing’s disease [28]. For this purpose, the so-called dexamethasone suppression test which can determine the origin of glucocorticoid hypersecretion (pituitary or adrenal origin) is often helpful. Pituitary adenoma is often associated with depression in cortisol and 17-hydroxycorticosteroid levels when a high dose of dexamethasone is administered because of negative feedback control, while adrenal tumors do not. Ultrasonography, angio-CT, and MRI can also be helpful in confirming the diagnosis. Ectopic glucocorticoid secreting tissues can be also detected using technetium 99 labeled octreotide scintigraphy examinations having receptors for somatostatin [24]. Arnaldi et al had recently described a detailed diagnosing algorithm [29].

### Anesthetic considerations

Hypercortisolism can be preoperatively controlled with adrenal enzyme inhibitors, such as ketoconazole, metyrapone, mitotane, or aminoglutethimide, given alone or in combination [30].

Adrenalectomy is also performed (open or laparoscopic) when a corticosteroid secretor adrenal hyperplasia is verified. Laparoscopic adrenalectomy remains gold standard. Cushing’s syndrome is associated with longer hospitalizations, more frequent major complications, and higher advanced care requirements, especially for bilateral adrenalectomy [31]. Preoperative optimization includes the control of hypercortisolism, hypertension, hyperglycemia, hypokalemia, and prevention of perioperative hypercoagulative state. The patients are often hypertensive and hypervolemic, under chronic antihypertensive drugs as well. All the drugs must continue till the morning of surgery except the angiotensin converting enzyme inhibitors (ACEI) and angiotensin II receptor blockers (ARB) because of their exaggerated hypotensive effects after the anesthesia induction. Spironolactone will decrease the potassium loss [32]. Hyperglycemia is found to be associated with increased mortality, higher rates of infections, and longer hospitalization [33-35]. Oral agents are generally discontinued before surgery [26], substituted with insulin. The goal is to maintain blood glucose levels within 120 - 180 mg/dL during the perioperative period [36]. American Association of Clinical Endocrinologists (AACE) and ADA have recommended target values to 140 - 180 mg/dL for ICU and 100 - 180 mg/dL as a target for the diabetic patients in medical and surgical wards [36]. The prevention of perioperative venous thromboembolism and PE can be realized using LMWH, lower-extremity compression devices [37], and early postoperative mobilization.

The anesthetic care consists in a detailed anesthetic plan, careful positioning and taping, and premedication technique. The anesthesiologist must take care during positioning and taping the patient, in order to prevent bone fractures and skin damages. Deep sedation must be avoided because of hypoxia risk and difficult airways. These patients are in increased risk for gastric aspiration, which can be prevented using metoclopramide, ranitidine, and sodium citrate. The detailed anesthetic plan includes possible difficult airway management, rapid induction of anesthesia, standard and/or invasive monitoring, large bore veins, central lines, and/or epidural catheter placement. Airway management may be difficult because of central obesity and proximal muscle weakness. The mask ventilation and endotracheal intubation may be difficult. Restrictive respiratory failure can be induced by obesity, associated with reduced functional residual capacity (FRC). Hypoventilation, atelectasis, and hypoxia may be the consequences of reduced FRC. These respiratory complications can be prevented by suitable preoxygenation, and appropriated extubation. Standard monitoring includes non-invasive blood pressure, temperature, end-tidal carbon dioxide, pulse oximetry, and electrocardiography. Invasive monitoring includes invasive blood pressure monitoring through an arterial catheter cannulation, and if necessary Swan-Ganz pulmonary artery catheter [14]. Large bore vein catheters, and central vein accesses are mandatory, in order to facilitate the fluids and drugs administration. The use of epidural anesthesia seems to be helpful [38] in controlling pain and reducing cardiac and pulmonary complications. Hydrocortisone succinate must be available in operating room in order to prevent a possible glucocorticoid deficiency. Aggressive pain treatment, early mobilization, hypertension and hyperglycemia control, and finally cortisol level monitoring are the essential postoperative problems that the anesthesiologist must deal with. As a conclusion, the controls of perioperative hypertension, hyperglycemia, hypokalemia, and cortisol blood level are hallmarks of the anesthesiologist’s role treating the Cushing patient. Table 3 summarizes all perioperative events.

**Table 3:** Perioperative Problems of Cushing’s syndrome Anesthetic Management

Cushing’s syndrome	Treatment
Preoperative period	
Cortisol inhibition	Adrenal enzyme inhibitors
Hypertension	Continue chronic therapy except ACEI and ARB
Hyperglycemia	Stop oral therapy and begin insulin regimen
Hypokalemia	Begin spironolactone and supplement potassium
Perioperative hypercoagulative state	LMWH, lower-extremity compression devices, and early postoperative mobilization
Intraoperative period	
Detailed anesthetic plan	General endotracheal anesthesia ± epidural
Positioning and taping	Careful and gentle positioning, avoid fractures
Premedication technique	Avoid deep sedation
Gastric aspiration risk	Drugs, rapid induction, Sellick maneuver
Airway management	Careful preoxygenation, ensure correct intubation
Venous access	Large bore peripherial and central venous catheters
Invasive monitoring	Radial artery cannulation, Swan-Ganz if required
Biochemistry tests	Close monitoring of glycemia, electrolytes, and pH
Postextubation respiratory failure	Awake extubation, close monitoring
Postoperative period	
Acute pain therapy	Aggressive treatment, systemic/epidural opioid
Biochemistry tests	Close monitoring of glycemia, electrolytes, cortisol and pH
Postoperative respiratory failure	Respiratory exercises, pain killers, mobilization
Venous thrombotic episodes	LMWH, early mobilization

## Pheochromocytoma

### General considerations

Pheochromocytoma presents the biggest challenge to the anesthesiologist compared with the other hormone secretion adrenal tumors. This disease is characterized by excess of catecholamine secretion inducing a sympathetic storm mostly presented by severe hypertension and arrhythmias. Pheochromocytoma can be adrenal or extrarenal (paraganglia), and can excessively secrete epinephrine, norepinephrine, and rarely dopamine. This tumor can also be associated with multiple endocrine neoplasia [39]. Ten percent of pheochromocytomas may be maligns, and 10% may be bilateral. It has been recently reported that 25-50% of deaths may occur during anesthesia induction [40].

The first signs suggesting pheochromocytoma are excessive sweating, headache, hypertension, arrhythmias, and palpitations. Imaging tools such as MRI and CT scan help find an adrenal tumor. The most important step in diagnosing pheochromocytoma is laboratory examinations that are summarized in Table 4.

**Table 4:** Sensitivity and Specificity of Pheochromocytoma Diagnosing Tests [10, 41]

Test/symptom	Sensitivity (%)	Specificity (%)
Vanillylmandelic acid	81	97
Catecholamine excretion	82	95
Metanephrine excretion	83	95
Abdominal CT scan	92	80
Paroxysmal hypertension, headache, sweating, tachycardia	90	95

### Anesthetic considerations

Anesthetic management of pheochromocytoma consists of several points: perioperative hemodynamic control, intraoperative control, and postoperative care. Generally perioperative hemodynamic control can be performed by anesthesiologist and endocrinologist. The mainstay therapy consists of combination of an α-adrenergic blocker and β-blocking agent. Short-acting, selective, competitive α1-adrenergic receptors blockers (doxazosin 2 - 6 mg daily) have been used in pheochromocytoma’s patients to prepare them for surgery. A potential advantage of competitive, selective α1-blockade is that, once the tumor has been resected and excess catecholamine release eliminated, α-adrenergic receptors return quickly to normal function, leading to less hypotension [41-43]. Phenoxybenzamine because of more severe and prolonged hypotension after the adrenal gland removal is usually stopped 24 - 48 h before surgery. Table 5 summarizes the differences between selective and nonselective α1-adrenergic receptors blockers [44].

**Table 5:** The Differences Between Phenoxybenzamine and Doxazosin

Phenoxybenzamine	Doxazosin
Non selective α1-adrenergic blocker	Selective α1-adrenergic blocker
Central signs present	No central signs (headache, nasal stuffiness)
β-blocker always necessary	β-blocker not always necessary
Prolonged and severe hypotension after adrenalectomy	No significant hypotension after adrenalectomy
Postural hypotension	No postural hypotension
Residual adrenergic blockade	No residual adrenergic blockade

Tachycardia must be treated using β-adrenergic blockers after the α-adrenergic blockade is effectively instituted. Other strategies include calcium blockers [45], clonidine, and magnesium sulfate [46]. An adequate adrenergic blockade is considered when the patient fulfills the following criteria: no hypertension (blood pressure over 160/90) during the last 24 h, no orthostatic hypotension, no change in ST-T wave, and no more than one premature ventricular contraction in 5 min.

Intraoperatively several points are to be considered. Standard and invasive monitoring is mandatory. Radial arterial catheter insertion must be performed before induction of anesthesia using local anesthetics such as EMLA. Large bore peripheral and central venous catheters are usually required to administer large amount of liquids and vasoactive drugs as necessary. The preoperative sedation must be judged case by case, but generally deep sedation ensuring airway management is strongly recommended. All drugs (pancuronium, ketamine, halothane, and desflurane) that can simulate the sympathetic system inducing hypertension and tachycardia are to be avoided. The anesthesia induction and endotracheal intubation must be smooth in order to avoid the increasing of the sympathetic tone. For this purpose the anesthesiologist can use different drugs [47-49] such as vasodilators (nitropruside, nitroglycerine, urapidil, and nicardipine), short-acting β-blocking drugs (esmolol), magnesium sulfate, and anesthetic drugs (remifentanil and propofol). Total intravenous anesthesia (combination of propofol and remifentanil as a continuous infusion) and dexmedetomidine [50] are modern options providing an adequate depth of anesthesia and blunting the sympathetic response during intubation and adrenal gland surgical manipulation. After the adrenal gland removal, a severe hypotension may occur. This hypotension is due to nonselective α-adrenergic blockers, deep anesthesia, and reduced blood volume (bleeding, diuretics, and preoperative uncorrected hypovolemia). Several vasopressors/inotropes can be used such as epinephrine, norepinephrine, dopamine, and vasopresine. Table 6 describes all vasoactive drugs used to control the hemodynamic during intraoperative period.

**Table 6:** Vasoactive Drugs Used During Pheochromocytoma’s Resection

Drug’s name	Dose	Comments
Vasodilator drugs (hypotensives)		
Propofol	2 - 2.5 mg/kg load, 25 - 75 μg/kg/min maintain	Local irritation, propofol infusion syndrome
Remifentanil	1 μg/kg load, 0.05 μg/kg/min maintain	Respiratory depression, hyperalgesia, vomiting
Dexmetedomidine	1 mg/kg load, 0.7 mg/kg/h maintain	Sedative effects
Nitroprusside	1 - 2 μg/kg/min	Severe hypotension, cyanide toxicity
Nitroglycerine	25 - 250 μg/min	Reflex tachycardia, methemoglobinemia
Nicardipine	5 mg/h	Braycardia, severe hypotension, cardiac blocks
Esmolol	5 - 10 mg/3 - 5 min bolus	AV block, bronchial hyperactivity
Labetalol	5 - 10 mg bolus	
Urapidil	10 - 15 mg/h	Severe hypotension
Clonidine	0.1 - 1.2 mg	Rebound hypertension, dry mouth
Magnesium sulfate	1 - 8 mg load, 1 - 4 mg/h maintain	Potentiates muscle relaxants
Vasoconstrictor drugs (hypertensives)		
Epinephrine	1 - 20 μg/min	Tachycardia
Norepinephrine	1 - 30 μg/min	Reflective bradycardia
Dopamine	5 - 10 μg/min	Tachycardia, arrhythmias
Phenilephrine	10 - 100 μg/min	Reflective bradycardia
Vasopressin	0.1 - 0.4 units/min	Myocardial infarction
Ephedrine	5 - 10 mg	None

As a conclusion, preoperative hormonal evaluation, preoperative hemodynamic control, smooth and gentle induction of anesthesia, modern anesthetic drugs, and strong intraoperative collaboration with surgical team, are the most important steps that can guarantee the successful management of pheochromocytoma’s resection.
